# Evaluation of electric vehicle performance using driving cycle clustering based on motor-inverter losses and efficiency

**DOI:** 10.1038/s41598-026-36663-3

**Published:** 2026-02-10

**Authors:** Khalil Abdelali, Bachir Bendjedia, Nassim Rizoug, Habib Kraiem, Aymen Flah

**Affiliations:** 1 Laboratory of Analysis and Control of Energy Systems and Power Network (LACoSERE), University of Amar Telidji, Laghouat, 03000 Algeria; 2 Laboratoire des Systémes et Energies Embarqués pour les Transports (ESTACA), S2ET Laboratory, 53061 Laval, France; 3 Center for Scientific Research and Entrepreneurship, Northern Border University, 73213 Arar, Saudi Arabia; 4 Applied Science Research Center, Applied Science Private University, Amman, 11931 Jordan; 5 ENET Centre, CEET, VSB-Technical University of Ostrava, 70800 Ostrava, Czech Republic; 6 Centre for Research Impact and Outcome, Chitkara University, Punjab University, Punjab, Rajpura, 140401 India

**Keywords:** Clustering, Driving Cycles, Electric Vehicles, Interior Permanent Magnet Synchronous Motor, Finite Element Analysis, Inverter Modeling, Energy science and technology, Engineering

## Abstract

This paper introduces the methodology for evaluating electric vehicle (EV) performance by integrating motor and inverter efficiency analysis with total losses assessment across representative driving cycle simulations. The proposed methodology integrates finite element analysis (FEA) of the Interior Permanent Magnet Synchronous Motor (IPMSM) with comprehensive modeling of inverter power electronics to accurately assess power electro-mechanical and conversion losses under realistic operating conditions. By considering the joint influence of motor behavior and inverter dynamics, the methodology enables a more accurate evaluation of EV performance across diverse driving cycles, offering a deeper understanding of efficiency variations and loss distribution throughout real-world operation. Additionally, the methodology is designed to minimize computational complexity while maintaining high accuracy for performance evaluations. Three standard driving cycles the Federal Test Procedure (FTP), the Worldwide Harmonised Light Vehicle Test Procedure (WLTP), and the New European Driving Cycle (NEDC) were selected to capture diverse driving scenarios. Initially, motor performance was simulated across all operating points within these cycles. Subsequently, a clustering technique was applied to condense the dataset without compromising accuracy, enabling a direct comparison between full cycle and clustered simulations. The study also evaluates the influence of two inverter types, insulated-gate bipolar transistor (IGBT) and metal-oxide-semiconductor field-effect transistor (MOSFET), on motor efficiency and losses. A case study on a 48-slot IPMSM integrated into an EV system illustrates the approach. Results show that clustering significantly reduces computational time while preserving essential performance characteristics. Moreover, inverter modeling reveals notable differences in efficiency and power losses between IGBT and MOSFET-based systems. These findings highlight the necessity of integrating motor and power electronics modeling for comprehensive EV design.

## Introduction

The global pursuit of sustainable mobility and the reduction of greenhouse gas emissions have accelerated the deployment of electric vehicles (EVs), driving the need for advanced and highly efficient traction motor technologies. As the demand for enhanced performance, power density, and energy efficiency continues to grow, optimizing traction motor design has become a central focus in electric drivetrain development^1^. Conventional design methodologies, primarily based on Finite Element Analysis (FEA), typically target performance optimization at a single operating point. However, such an approach is unable to capture the diverse operating conditions of real-world driving scenarios, thereby constraining improvements in efficiency and torque performance across the complete operating envelope^2^. Consequently, there is a growing emphasis on holistic design frameworks that integrate power electronics, control systems, and thermal effects to ensure robust performance under variable load and speed conditions^3^.

Traditional FEA-based motor design methods^4–9^ frequently neglect the impact of power electronics–particularly the inverter–despite its considerable influence on the overall system efficiency. The inverter governs the current supplied to the motor, directly affecting torque generation, switching losses, and total powertrain performance. Excluding inverter dynamics during the design phase can lead to overestimated efficiency and inaccurate prediction of operating losses, particularly in high-power-density EV applications. Therefore, incorporating inverter effects within the electromagnetic and optimization workflow is essential to ensure the accurate representation of real-world drive performance.

Recent research has made progress in improving electric machine evaluation by considering full driving cycles^2,8,10–13^. Advanced techniques, such as k-means clustering and the Energy Center of Gravity (ECG) method, have been employed to identify representative torque–speed points that reduce computational cost while maintaining overall performance characteristics. These data reduction methods allow for faster design iterations and more efficient evaluation of motor operation across variable conditions. However, most existing studies that employ such clustering or summation approaches primarily focus on the electromagnetic domain and omit the influence of inverter behavior or the coupled dynamics of the full powertrain. As a result, the accuracy of system-level performance estimation remains limited when extended to realistic EV operation.

**Fig. 1:**
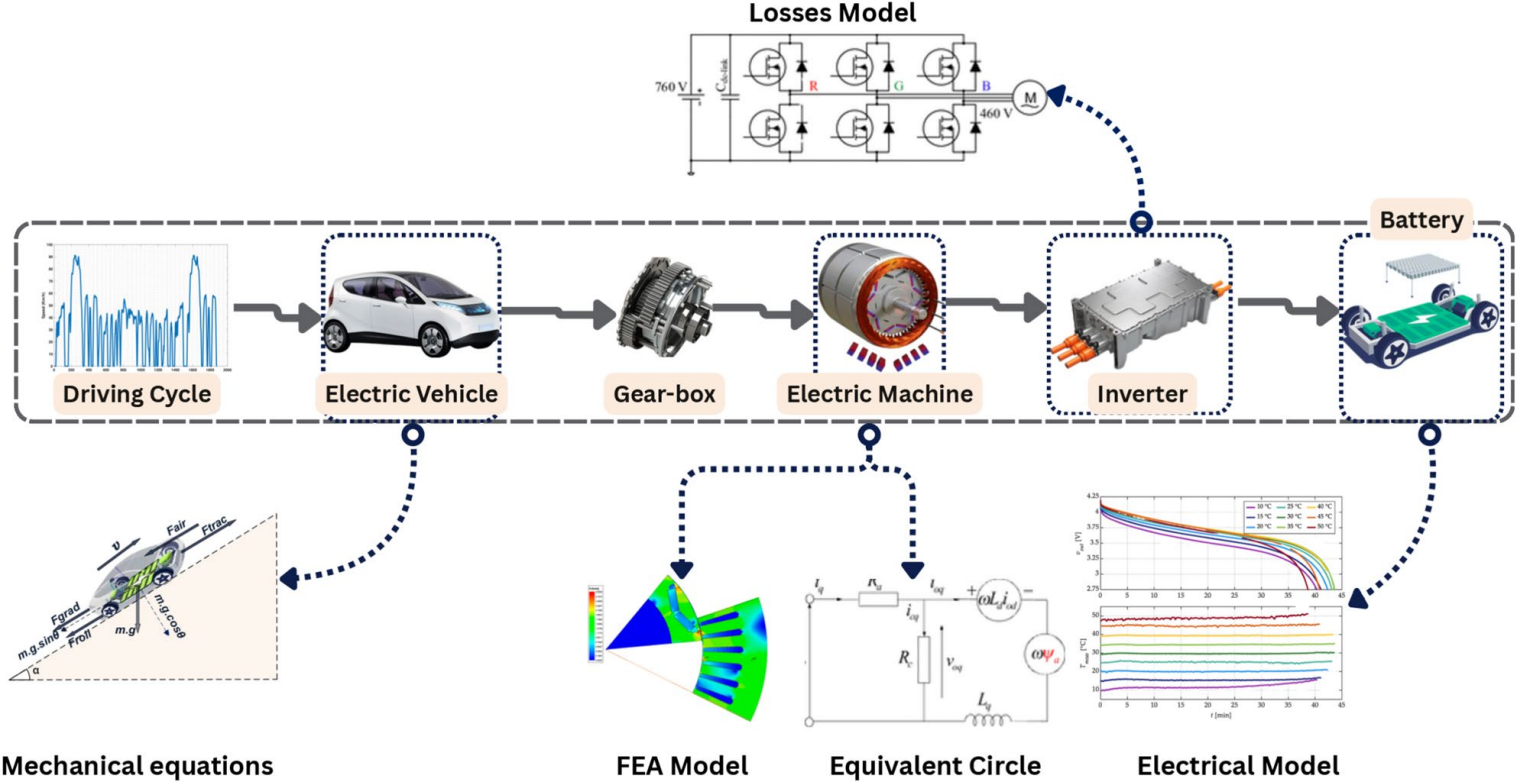
Methodological framework for powertrain performance evaluation.

In this context, the present study proposes a novel integrated optimization and simulation framework that addresses these limitations. The proposed approach efficiently represents the entire driving cycle through a weighted summation of representative operating points, which significantly reduces computational time while maintaining accuracy comparable to full-cycle simulations. Unlike previous studies, this framework explicitly incorporates inverter dynamics and full powertrain interactions, allowing for a more realistic assessment of torque production, system losses, and overall efficiency. The integration of MATLAB-based inverter modeling with Ansys Maxwell FEA ensures that both electromagnetic and converter effects are simultaneously evaluated, providing a complete picture of drivetrain performance. The proposed methodological framework, illustrated in Fig. 1, begins with an analysis of the selected driving cycles to evaluate how vehicle mass and power demand influence drivetrain performance. An EV dynamics model is employed to compute the wheel torque required to follow a given speed profile and road slope over time, using classical mechanical equations. In this study, a fixed gear ratio is adopted for the transmission system, which scales the torque and speed between the electric motor and the wheels, a common configuration in electric vehicles. This ratio reduces the torque required from the motor while increasing its operational speed^14^.

Although the tractive effort is applied at the wheels, the main focus of the work is the electric motor. Therefore, the calculated vehicle force is translated back to motor shaft torque using the wheel moment arm, enabling accurate determination of the motor’s torque–speed operating points. To supply the motor with the electrical power required to meet these operating conditions, the required voltage and current are delivered through an inverter powered by the high-voltage battery.

In this study, Fig. 1 provides the full powertrain context. However, detailed electrochemical battery modelling is not required for the traction-focused analysis. The framework primarily aims to extract the motor torque–speed operating points from the driving cycles. After computing the tractive torque and converting it through the fixed gear ratio, the motor envelope is assessed. Simulating full battery–inverter switching dynamics and motor over the entire driving cycle would require prohibitive computational time. Therefore, the battery is represented as an equivalent constant DC voltage source, which is sufficient for supplying the inverter in this context. Battery electrochemical behavior (OCV–SOC characteristics, impedance, etc.) is outside the scope of the traction efficiency and loss evaluation and is thus not modeled.

We acknowledge that a complete whole-vehicle model would incorporate additional components such as the on-board charger, auxiliary electrical loads, and other vehicle subsystems. However, this study focuses specifically on the traction system for several reasons: the on-board charger is inactive during driving cycles, auxiliary loads operate on a separate 12 V network electrically isolated from the high-voltage traction system, and their power consumption is negligible relative to traction power, exerting minimal influence on motor–inverter efficiency. Consistent with practices reported in the literature over the last three decades, auxiliary devices are often excluded from EV loss assessments^15,16^, as including them would require modeling the entire driving cycle with all switching events, necessitating high temporal resolution and substantial computational resources. This approach ensures that the analysis remains both focused and computationally feasible while capturing the dominant contributors to traction system losses.

###  Objectives and contributions

In conventional electric motor design optimization, the principal objective has often been to maximize efficiency under rated operating conditions, primarily constrained by the considerable computational cost of finite element analysis (FEA) simulations, as reported in^17^. In practical vehicular applications, however, electric machines operate across a broad continuum of torque and speed conditions that vary dynamically with the driving cycle. An Interior Permanent Magnet Synchronous Motor (IPMSM) cannot sustain its peak efficiency throughout this entire operational envelope. Consequently, a broader and more representative optimization framework is required one that evaluates performance across multiple operating points to ensure high efficiency under realistic, time-varying torque and speed demands.

Conventional design and simulation methodologies frequently assume a ideal sinusoidal current excitation. In reality, the motor is supplied by a voltage-source inverter that introduces harmonic distortions into the three-phase excitation waveform^18^. These inverter-induced harmonics, commonly referred to as time harmonics, deteriorate machine performance by amplifying torque ripple, mechanical vibration, and additional harmonic losses^19^. It is important to note that even when advanced control algorithms are applied through co-simulation within the FEA environment, performing a complete design optimization of the IPMSM remains computationally prohibitive due to the intensive numerical requirements of high-fidelity finite element models^20^. Therefore, incorporating inverter excitation models that explicitly include harmonic effects is essential to accurately predict motor behavior. Moreover, drive-cycle-based analyses are indispensable for evaluating global efficiency and dynamic response under conditions that closely replicate real-world operation.

The main contributions of this research are highlighted as follows:

- Enhancing the practical assessment of electric vehicle drives by integrating the motor, inverter, and drive-cycle dynamics into a unified powertrain framework, enabling accurate performance evaluation through finite-element analysis (FEA) under realistic operating conditions.
- Providing a comparison between full drive-cycle simulation and the representative clustering method, demonstrating that the reduced set of operating points preserves the dynamic characteristics of the entire cycle. This enables optimization to be performed over a wide range of drive cycle conditions rather than a single operating point, ensuring that the resulting designs remain valid across the full operational envelope of the vehicle.
- Integrates realistic inverter excitation models, based on IGBT and MOSFET into the IPMSM finite element analysis (FEA) simulation framework, facilitating the assessment of motor performance under inverter-fed situations. This study quantifies the impact of harmonic distortions from inverters on torque ripple, efficiency, and electromagnetic losses by comparing them to ideal sinusoidal excitation, highlighting the importance of considering inverter-induced harmonics in precise motor design and performance evaluation.
- Modeling inverter switching and conduction losses, for both IGBT- and MOSFET-based converters within the powertrain framework, enabling a comprehensive drive-cycle analysis that captures the inverter’s contribution to total power consumption. This extends the evaluation from a motor-only loss model to a unified motor-inverter loss assessment at the powertrain level.
- Performs a comprehensive assessment of computational efficiency by comparing entire drive-cycle simulation durations with those derived from clustered representative points, both excluding inverters and using integrated inverter models. This research estimates the computational load imposed by inverter dynamics and illustrates the significant efficiency achieved by the clustering-based reduction of operating points.

### Organization of Study

The paper is organized to progressively develop the modeling, analysis, and evaluation framework for the proposed IPMSM drive cycles investigation. Section Methods presents the foundational aspects of vehicle dynamics, emphasizing the influence of power demand and vehicle mass on drivetrain modeling accuracy, and analyzing the speed profiles and load characteristics extracted from three representative driving cycles. Corresponding machine parameters are derived through finite element analysis (FEA) using Ansys Maxwell. Section Clustering techniques for drive-cycle data representation outlines the methodological framework, introducing the Enhanced Clustering Grouping (ECG) technique for efficient representation and reduction of drive-cycle data, and describing the implementation of the Maximum Torque Per Ampere (MTPA) control strategy used to define current excitation values for the clustered representative operating points. Section Performance analysis without inverter extends the analysis to motor loss and efficiency evaluation across the complete operating range of the driving cycle, comparing full-cycle simulations with the clustering-based approach to highlight the trade-offs between computational cost, modeling accuracy, and resource efficiency. Finally, Section Performance analysis with inverter current excitations examines the influence of inverter dynamics on system performance by integrating IGBT- and MOSFET-based inverter topologies, modeled in MATLAB/Simulink and coupled with the Ansys Maxwell environment, to assess their impact on efficiency, torque ripple, and total losses throughout the driving cycle, thereby providing a comprehensive system-level performance assessment.

## Methods

### Model of the electric vehicle

Vehicle dynamic models are essential for forecasting performance and enhancing system designs in automotive engineering. These simulations allow engineers to evaluate vehicle performance under various operating scenarios, ensuring that the final models fulfill real-world performance standards.

Vehicle dynamic simulations determine the motor torque and speed requirements under different driving scenarios. These simulations are essential for forecasting vehicle performance and behavior in various settings. These standards help engineers understand how numerous elements affect vehicle functioning^21,22^.

**Fig. 2:**
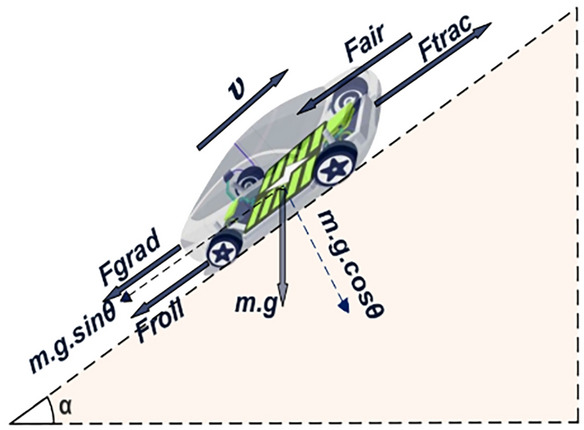
Visualization of force modelling at the vehicle level.

Figure 2 provides a comprehensive overview of fundamental factors affecting vehicle dynamics, highlighting the forces and circumstances to consider in designing and optimizing vehicle systems.

The car’s resistance increases with road gradient, increasing gravitational force as it climbs^23^. Equation 1 calculates the vehicle’s force from drag, friction, acceleration, and gravity. Understanding and improving vehicle behavior requires understanding the resultant force^24^.

1 $$\begin{aligned} F_{T}=F_{air} + F_{roll} + F_{grade} + F_{Acc} \end{aligned}$$

The relationship between vehicle speed ($\upsilon$) and mechanical force on the tire produces mechanical power. The product of these two variables shown in Eq. 2 illustrates the inherent relationship between speed and force in power transmission. This relationship produces power for efficient vehicle propulsion^25,26^.

2 $$\begin{aligned} P_{m}= \upsilon \, F_{T} \end{aligned}$$

This project examines a small, two-passenger electric vehicle fitted with a distributed drivetrain. The dynamic vehicle model was derived from the Bluecar Beloré, a well-renowned micro-sized electric vehicle noted for its unique powertrain and energy-efficient performance^23,27^. Table 1 provides a comprehensive overview of the technical specifications and features of the electric vehicle that have been included in the model.

**Table 1:** Basic Blue Car Beloré Micro-Size EV specifications^28^.

Parameter	Value	Unit
Vehicle mass	860	kg
Tire diameter	0.33	m
Rolling resistance coefficient	0.008	–
Coefficient of aerodynamic resistance	0.3	–
Gravitational acceleration	9.81	m/s^2^
Frontal area	2.75	m^2^
Differential gear ratio	3	–

### Driving cycle selection

**Fig. 3:**
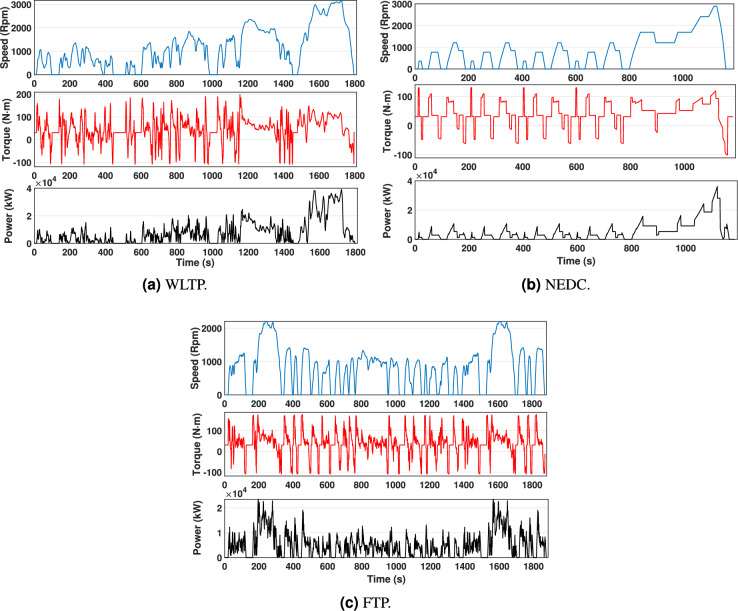
Analysis of EV performance over different driving cycles.

The most widely recognized driving cycles include the Worldwide Harmonized Light Vehicles Test Procedure (WLTP), the New European Driving Cycle (NEDC), and the Federal Test Procedure (FTP), which are standard methodologies for evaluating vehicle emissions, fuel economy, and overall performance. The WLTP and NEDC are primarily used in Europe, while the FTP cycle is widely adopted in the United States for assessing light-duty vehicle efficiency under diverse driving conditions. These cycles serve as benchmarks for regulatory testing, ensuring that vehicles comply with environmental and performance standards^21^. This study specifically examines the WLTP, NEDC, and FTP driving cycles, as they represent key global standards for passenger vehicles. Their selection is based on their widespread adoption in the automotive industry and their relevance to both manufacturers and consumers. By simulating real-world driving conditions, these cycles provide critical insights into EV efficiency, range, and energy consumption. Figure 3 illustrates these standardized cycles, serving as a reference for analyzing EV performance across different driving scenarios.

The study, based on these driving cycles, eliminates road inclination. Equations 3 and 4 detail the calculations for motor load torque values $T_L$ and the rotational speeds of the rotors $\omega _r$. The gear ratio is denoted by $n_g$, and the efficiency of the gear is represented by $\eta _{gear}$. Equation 3 defines the relationship between the rotational speed of the motor, $\omega _{mot}$, and the rotational speed of the wheels, $\omega _{wheel}$, taking into account the gear ratio^29^.

3 $$\begin{aligned} & \omega _{mot} = \frac{\omega _{wheel}}{3.6 \, r_{wheel}} \cdot n_g \end{aligned}$$

4 $$\begin{aligned} & T_{mot} = \frac{F_{T}\, r_{wheel}}{n_g.\eta _{gear}} \end{aligned}$$

A constant gear ratio is employed in electric vehicle design to simplify torque delivery across various driving conditions. This study utilizes a dynamic model for EVs developed in MATLAB to generate motor torque, speed profiles, and power characteristics, ensuring a comprehensive analysis of drivetrain performance. Figure 3 illustrates the specified vehicle’s simulated power requirements and torque outputs across the three driving cycles, offering helpful details about its operational efficiency.

**Fig. 4:**
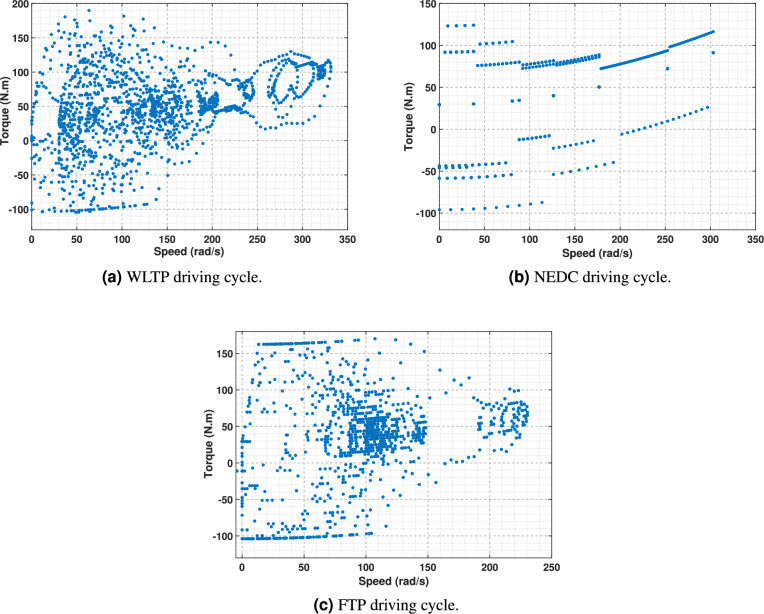
Torque vs. Speed points over different driving cycles.

The drive-cycle loading characteristic can be effectively analyzed on a torque-speed plane, as illustrated in Fig. 4. This graphic eliminates the loading condition’s temporal dependence and instead illustrates the instantaneous output torque and speed experienced by the electric motor at each discrete sample point of the driving cycle. This graph is a helpful technique for establishing motor design objectives for the maximum torque and speed possibilities of an electric machine according to practical driving conditions^30^.

To accurately evaluate the machine’s performance during the driving cycle, it is necessary to ensure the motor’s efficiency at each load point within the torque and speed range, as shown in Fig. 4. Nevertheless, this procedure might be computationally intensive due to the substantial quantity of individual sample points that must be analyzed. In order to tackle this difficulty, statistical data-mining methods are utilized to efficiently decrease the amount of data to a smaller, more controllable set of representative points. The selection of these points is performed meticulously to preserve the core characteristics of the full dataset, ensuring the retention of critical data. This allows for a comprehensive evaluation of machine behavior throughout the entire driving cycle, with the requirement to analyze each data point. As a result, this detailed analysis can be effectively integrated into an optimization method^31^.

### Energy distribution on the torque-speed plane

Motor performance and efficiency are directly influenced by energy consumption during loading. Inefficiencies in this process can lead to excessive battery power usage, ultimately reducing the vehicle’s range and overall performance under real-world driving conditions^24^.

In this study, energy consumption is assessed by integrating power output over time, offering a comprehensive evaluation of the total energy utilized throughout an entire driving cycle. The analysis relies on essential data, including vehicle speed, obtained from three standardized driving cycles WLTP, FTP, and NEDC. By analyzing the speed profiles of these driving cycles and the corresponding power exerted by the vehicle, the total energy consumption is determined. This calculation captures the dynamic interaction between the powertrain and the vehicle’s operating demands over time. The mathematical formulation of this energy computation is presented in Eq. 5. The equation for energy is as follows:

5 $$\begin{aligned} E_{ij} = \Delta t \, P_{\text {out},ij}(t) \, dt \end{aligned}$$

**Fig. 5:**
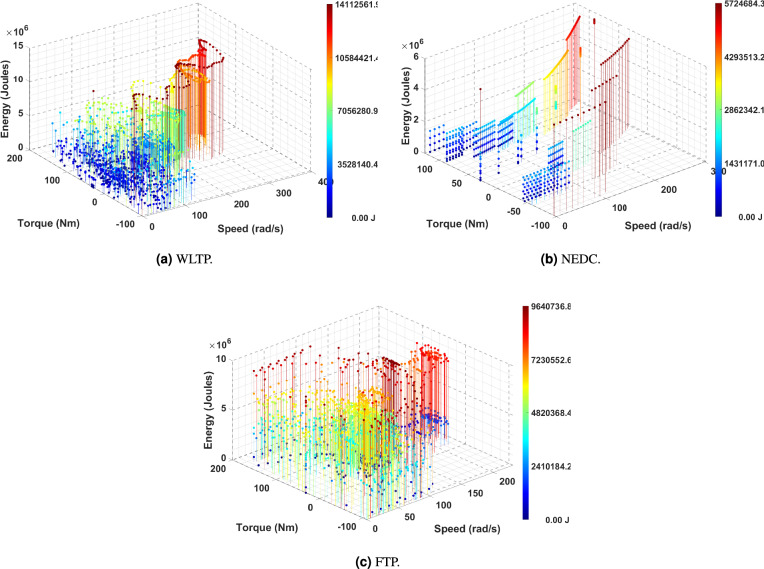
Energy distribution during the torque-speed plane.

where:

- $$E_{ij}$$: Energy ( Joules )
- $$P_{out,ij}$$: Power ( Watts )
- $\Delta t$: Interval of time ( s )

The motor energy, $E_{\text {motor}}$, is computed by summing individual power measurements over the entire operational period. Additionally, the energy associated with each load point on the torque-speed plane is determined by multiplying the frequency of occurrence within the driving cycle by the corresponding power level^24^. This methodology enables a detailed examination of energy distribution across the torque-speed plane, as illustrated in Fig. 5, providing critical insights for optimizing motor efficiency in regions with high energy demands.

## Clustering techniques for drive-cycle data representation

The procedures’ clustering methods are used to reduce load data into a minimal number of points that retain and reflect the characteristics of the entire dataset. This method improves computational efficiency when evaluating a machine’s performance over a complete driving cycle, thus allowing for the integration of these assessments into an optimization algorithm^31^.

### K-means clustering

Centroid-based partitional clustering using k-means is prominent in data mining and classification. This method has been utilized to cluster electric motor driving cycles^32–36^. The procedure involves separating data points into k-means clusters and choosing the number of clusters based on the SSE method. Data points go to the cluster with the closest centroid. k-means aims to minimize the objective function, which evaluates the distance between data points and cluster centroid over n data points and k clusters, as illustrated in^23^. The main steps are iteratively updating centroids and reassigning data points based on their proximity to the next centroid.

#### Clustering selection

Excessive clustering in machine learning may lead to the overprediction of a dataset. An increase in clusters results in decreased accuracy for new value predictions owing to outliers within the dataset. However, applying this machine-learning technique to a static dataset typically enhances representation accuracy by increasing the number of clusters in K-means. Therefore, selecting an optimal number of clusters to represent the data involves a delicate balance between representational quality and processing efficiency.

**Fig. 6:**
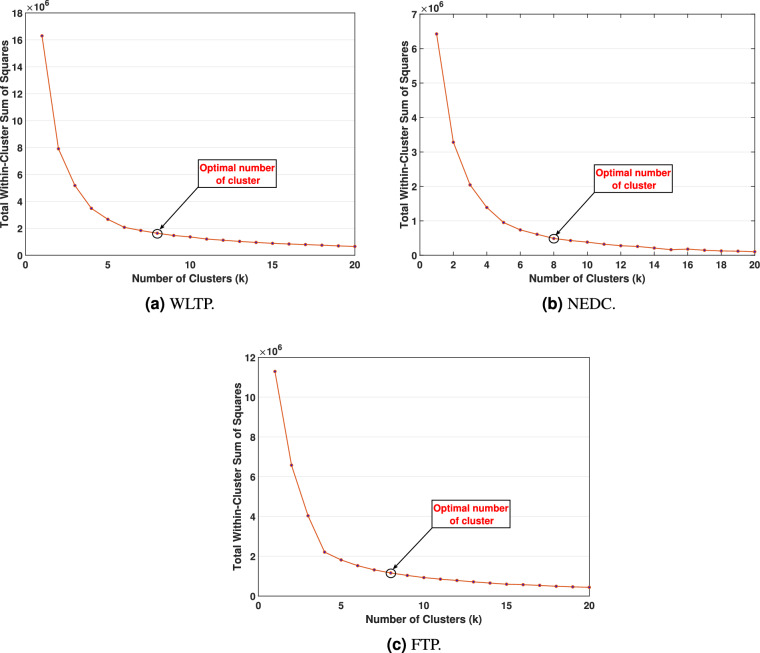
Optimal cluster selection using the SSE method.

The optimal number of clusters is determined using the Sum of Squared Errors (SSE) method, which evaluates the variance within clusters to ensure effective data representation. The SSE curves in Fig. 6 were specifically used to determine the appropriate number of clusters. The following equation illustrates the SSE calculation^37^:

6 $$\begin{aligned} {SSE=\sum _{i=1}^{k}\sum _{x\epsilon m_i}{dist{(x,\ m_i)}^2}} \end{aligned}$$

As expected in K-means analysis, the SSE value decreases exponentially toward zero as the number of clusters approaches the total number of data points, creating the characteristic “elbow” pattern. This elbow marks the point beyond which additional clusters offer only negligible improvement in representation. For all three driving cycles (WLTP, NEDC, FTP), this inflection clearly occurs at k = 8, indicating that eight clusters capture the essential variability of the driving cycles while avoiding unnecessary computational cost. The physical significance of this point lies in the fact that further increasing k does not introduce new meaningful operating conditions but only increases simulation time.

**Fig. 7:**
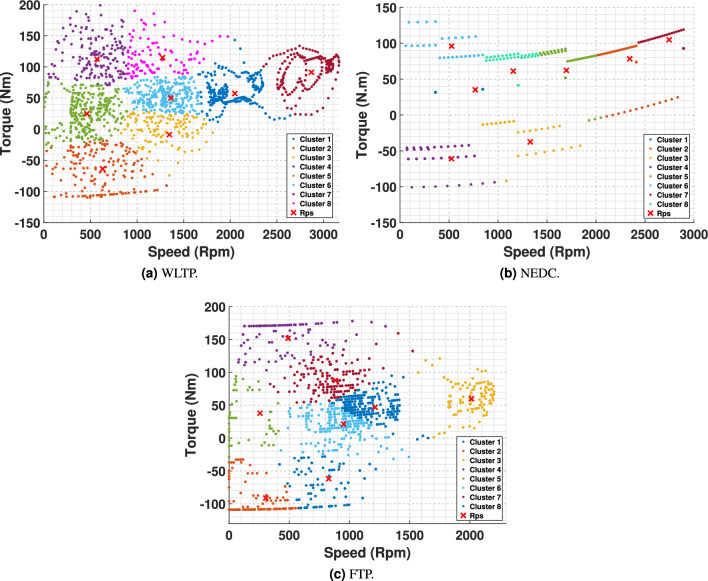
Clustering result for torque-speed load data.

This technique offers a revised allocation of clusters and distribution of representative points throughout the dataset, as shown in Fig. 7, where the different colors indicate diverse clusters and the noticeable red dots indicate the centroids. The torque-speed points are derived from the vehicle dynamics data presented in Section Methods.

### Addressing energy significance in clustering algorithms

The k-means clustering method only uses the torque and speed data to group the data, so it doesn’t take into account the energy significance of the points in a given cluster. Therefore, it is important to recognize that proposing a new approach could improve the situation by achieving the optimal distribution and weighted significance of the representative data points.

#### ECG clustering technique

By integrating the k-means clustering method with the Energy Centre of Gravity (ECG) technology, it is possible to obtain the most optimal representation for any given number of clusters on each driving cycle. This technique accurately represents data points for each count of driving cycle clusters^33,34^. As per Eq. 7, the energy of each data point, $E_{ij}$, is scaled to the total energy of the cluster, $E_i$. This normalized value determines the weighting of the ECG. The sample data points are obtained by calculating each cluster’s weighted averages of torque and speed. The selection of the locations $\omega _{mc_i}$ and $T_{mc_i}$ is based on their energy importance. Equations 8 and 9 demonstrate how these locations accurately and explicitly represent clustered data^31^^38^. This approach guarantees that the clustering procedure integrates data distribution and energy importance.

7 $$\begin{aligned} & E_{i} = \sum _{j=1,2,\dots }^{N_i} E_{ij} \end{aligned}$$

8 $$\begin{aligned} & \omega _{mci} = \frac{1}{E_i} \sum _{j=1,2,\dots }^{N_i} E_{ij} \omega _{mij} \end{aligned}$$

9 $$\begin{aligned} & T_{ci} = \frac{1}{E_i} \sum _{j=1,2,\dots }^{N_i} E_{ij} T_{mij} \end{aligned}$$

### Weight assignment

Assigning appropriate weights to each Representative Point (RP) in the design investigation process is essential for accurately capturing its impact on overall system performance. These weights ensure that each RP is considered in proportion to its significance, enabling a more precise analysis of energy distribution and efficiency. The weighting process accounts for two key factors: the energy throughput at a given operating point and its frequency of occurrence throughout the driving cycle.

The determination of RP weights is typically based on the ratio of energy consumed by all points within a specific cluster to the total energy consumption across all clusters. As formally expressed in Eq. 10, this method ensures that weights accurately reflect the energy intensity of different regions within the driving cycle, emphasizing areas of higher energy demand. By prioritizing these high-energy regions, the investigation process can effectively enhance efficiency and optimize performance in the most critical operational conditions.

10 $$\begin{aligned} E_{w,i} = \frac{E_i}{\sum _{j=1}^k E_j} \end{aligned}$$

Table 2 summarizes the final values for each identified cluster to assess performance across the three standardized driving cycles: WLTP, NEDC, and FTP. The table presents detailed numerical data for representative operating points, including torque and speed, as well as the normalized energy contribution of each point. This provides valuable insight into the energy distribution and operational behavior of the IPMSM within the torque–speed domain.

**Table 2:** Representative operating Points on different driving cycles.

Driving Cycle	Speed (rad/s)	Torque (N$\cdot$m)	Weighted Energy
WLTP	214.49	57.30	0.260
	66.04	-63.79	0.078
	48.35	24.76	0.079
	300.49	91.25	0.270
	142.69	50.07	0.120
	141.04	-8.71	0.077
	133.40	114.97	0.058
	59.71	112.47	0.050
NEDC	80.41	35.10	0.085
	245.51	78.26	0.172
	139.18	-37.52	0.023
	55.00	-60.96	0.038
	177.77	62.17	0.233
	55.08	95.99	0.099
	287.59	104.79	0.151
	121.00	61.13	0.200
FTP	92.49	87.59	0.116
	127.09	46.83	0.239
	51.37	151.71	0.094
	26.96	37.75	0.045
	86.68	-61.83	0.071
	99.60	21.27	0.208
	31.95	-91.23	0.084
	210.91	59.90	0.141

## Performance analysis without inverter

### Motor model

**Fig. 8:**
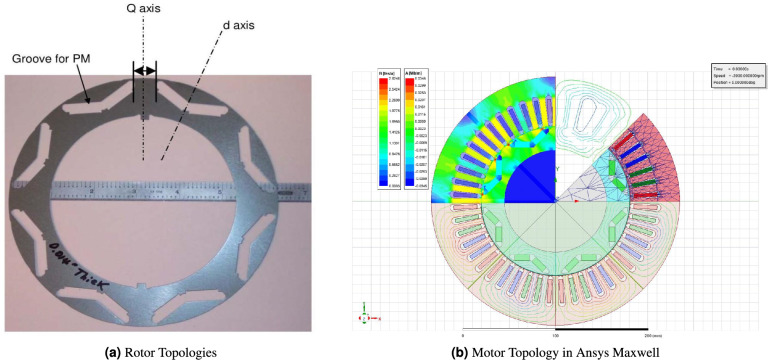
Cross-sectional view of the IPMSM model.

The IPMSM is widely recognized as a highly suitable choice for automotive applications, owing to its superior efficiency, high power density, favorable torque-to-current ratio, and broad operational speed range^39^. Figure 8 presents a cross-sectional view of the IPMSM, illustrating the flux density distribution under operating conditions of 245 Nm torque and 175 A RMS per phase. This motor is utilized as a benchmark to evaluate the methodology developed in this study. The specifications of the IPMSM used in the analysis are summarized in Table 3.

**Table 3:** Characteristics of IPMSM.^40^.

Specification	Value	Unit
Rated power	50	kW
Rated / Maximum torque	245 / 400	N.m
Rated current	176	A
Base speed	1500	rpm
Number of poles / slots	8 / 48	–
Outside / Inside stator diameter	269.2 / 161.96	mm
Outside / Inside rotor diameter	160.4 / 110.64	mm
Air gap length	0.75	mm
Lamination length	82.83	mm
Type of iron (Stator / Rotor)	M19-29G	–
**Iron Loss Coefficients**		
Eddy current loss coefficient, $k_c$	0.38626	–
Hysteresis loss coefficient, $k_h$	184.23	–
Excess loss coefficient, $k_e$	0.27023	–

###  MTPA current defining at operational locations

The primary goal of this section is to calculate the optimal values of the direct axis current ($i_d$) and the quadrature axis current ($i_q$) by using the Maximum Torque per Ampere (MTPA) approach utilizing Eqs. 12 and 13. The objective is to enhance the voltage within the operational limit while minimizing the current needed to provide the specified torque at a given speed. The instantaneous electromagnetic torque generated in a permanent magnet synchronous machine may be expressed as :

11 $$\begin{aligned} & T_{e}=\frac{3}{2}\, p \,(i_q \, \psi _d - i_d \, \psi _q) \end{aligned}$$

12 $$\begin{aligned} & i_{d}= - I_s \, \sin {(\gamma )} \end{aligned}$$

13 $$\begin{aligned} & i_{q}=I_s \, \cos {(\gamma )} \end{aligned}$$

To conduct this analysis, we examine the torque profiles for various levels of stator current, spanning from $0^\circ$ to $90^\circ$ current control angles $(\gamma )$, while running at a significantly greater speed than the base speed.

The torque in this area is constrained by both voltage and current limits. They are making it essential to ensure that the machine operates safely and efficiently. Therefore, it is difficult to determine the exact current magnitude and control angle that meet the voltage limit and generate torque. Ensuring a harmonious equilibrium between current and voltage is vital in order to safeguard against any potential damage or reduction in the effectiveness of the equipment. The Eqs. 14 and 15 represent the terminal voltage and current equations of the IPMSM when it is in a stable condition^13^.

14 $$\begin{aligned} & V_{max} \geqslant \sqrt{V_d^2 + V_q^2} = \frac{2}{\sqrt{3} } V_{dc} \end{aligned}$$

15 $$\begin{aligned} & I_{m} \leqslant \sqrt{i_d^2 + i_q^2} \end{aligned}$$

**Fig. 9:**
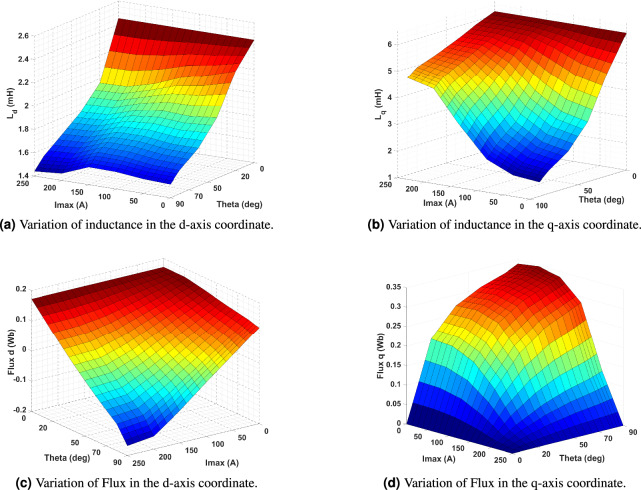
Results FEA maps for IPM motor.

For defining all the data for MTPA, the current in ANSYS MAXWELL is utilized using an ideal sinusoidal excitation. Figure 9a and 9b illustrate the d-q inductances of the analyzed machine, obtained using the proposed model. Notably, in an interior V-shaped permanent magnet machine, the q-axis inductance ($L_q$) is greater than the d-axis inductance ($L_d$). Furthermore, the flux linkages exhibit dependency on the direct and quadrature axis currents ($i_d$ and $i_q$), meaning they vary in response to the stator phase current ($I_s$) and the control angle ($\gamma$). This variation is depicted in Fig. 9c and 9d, highlighting the influence of these parameters on the machine’s electromagnetic behavior.

The d- and q-axis flux linkage data are utilized to compute motor torque and required speed at each operating point, as summarized in Table 4. which is crucial for implementing the MTPA strategy to improve motor efficiency and performance. By developing flux linkage curves for both axes, we gain insights into the motor’s magnetic characteristics, enabling a more accurate representation of its behavior under various operating conditions.

Accurate torque calculations require a detailed analysis of the d-q flux correlation. Examining these relationships allows for precise torque estimation across different load conditions. Additionally, motor inductance values are derived from the flux linkage data, further refining the torque computation process. To determine the permanent magnet flux ($\psi _{PM}$), FEA is conducted in Ansys Maxwell. The results indicate that $\psi _{PM}$ is approximately 0.1724 Wb, a critical parameter that directly influences the motor’s electromagnetic properties and overall performance.

**Table 4:** MTPA data results related to clustering points.

Drive cycle	$\mathbf {I_m\,(A)}$	$\mathbf {Angle\,(^\circ )}$	$\mathbf {i_d\,(A)}$	$\mathbf {i_q\,(A)}$	$\mathbf {L_d\,(mH)}$	$\mathbf {L_q\,(mH)}$
WLTP	45.0	26.35	-19.98	40.32	2.2723	5.3058
	49.5	148.90	-25.57	-42.38	2.4026	5.0556
	20.5	17.76	-6.25	19.52	2.4868	5.9164
	68.5	32.66	-36.96	57.67	2.0949	4.6357
	39.7	26.36	-17.62	35.57	2.3156	5.5059
	7.4	155.60	-3.06	-6.74	2.6172	5.9418
	84.4	36.10	-49.72	68.20	2.0054	4.2997
	82.7	36.10	-48.72	66.82	2.0141	4.3448
NEDC	28.5	22.92	-11.10	26.25	2.4143	5.2806
	59.6	31.51	-31.15	50.81	2.1534	4.8897
	30.3	152.00	-14.22	-26.75	2.5326	5.6359
	47.5	149.10	-24.39	-40.76	2.4179	5.1228
	48.4	28.65	-23.20	42.47	2.2399	5.2221
	71.7	33.23	-39.29	59.97	2.0754	4.5572
	77.6	34.38	-43.82	64.05	2.0413	4.4241
	47.7	27.50	-22.03	42.31	2.2480	5.2244
FTP	66.0	32.08	-35.06	55.92	2.1109	4.6967
	37.3	26.93	-16.89	33.25	2.3330	5.5958
	109.7	39.53	-69.83	84.60	1.8925	3.8342
	30.6	21.77	-11.35	28.42	2.4037	5.7538
	48.1	149.60	-24.34	-41.49	2.4136	5.0917
	17.7	16.61	-5.06	16.96	2.5079	5.9454
	68.4	144.60	-39.62	-55.75	2.2671	4.5460
	46.8	28.07	-22.02	41.29	2.2537	5.2704

### Losses analysis

Accurately quantifying losses is essential for optimizing machine efficiency. Understanding these losses is essential for effectively managing the cooling system of the electric machine since limiting the occurrence of hot spots is vital for the machine’s durability and continuous power generation.

The stator in an IPMS is mainly affected by copper and core losses, whereas the rotor is mainly affected by core, magnet, and mechanical losses. Dividing the magnets into segments can reduce eddy current losses. Using a laminated core with thinner layers helps mitigate stator and rotor core losses by minimizing eddy current losses, which become more pronounced at higher frequencies. The total losses $P_{loss}$ can be determined using the following equation:

16 $$\begin{aligned} P_{loss}= P_{cu} + P_{core} \end{aligned}$$

Copper and iron losses were evaluated for each operating point using transient finite-element simulations in Ansys Maxwell 2D, where average loss values were extracted over a full electrical period and iron losses were computed using the constant material loss coefficients provided in the Ansys database Table 3. Magnet losses, although available in Maxwell, were intentionally excluded because their accurate calculation requires a significantly finer mesh within the magnets, which would substantially increase simulation time without materially affecting the comparative outcomes. Similarly, mechanical losses, including windage and bearing friction, were excluded, as previous studies indicate they generally represent only $1\text {-}3 \%$ of the rated power for traction-type IPMSMs, rendering their impact negligible compared to the dominant copper, iron, and inverter-induced losses analyzed in this study^41^. The analysis prioritizes the most significant loss components while ensuring computational efficiency across various operating points.

### Efficiency analysis using clustering method

This study applies FEA in Ansys Maxwell to systematically evaluate key performance parameters, including average torque, spatial harmonic torque ripple, and flux density distribution within the stator tooth and yoke regions. This approach enables precise quantification of core losses, which is essential for improving energy efficiency in electric vehicle applications. Furthermore, FEA facilitates a comprehensive current scan along the d- and q-axes, allowing for the precise determination of current excitations necessary to achieve the target speed and torque at various RPs identified through the clustering method. To further enhance motor performance, the MTPA algorithm is integrated into the simulations, effectively minimizing power losses while maximizing torque generation.

Motor efficiency at each representative point is evaluated using Eq. 17, where efficiency ($\eta$) is defined as the ratio of mechanical output power to total electrical input power after accounting for copper and iron losses. The overall efficiency over the entire drive cycle can also be evaluated using Eq. 18, based on weighted efficiency ($\eta _{w}$) extracted from the ECG method.

17 $$\begin{aligned} & \eta = \frac{T_e .\, \omega }{T_e .\, \omega + P_{cu} + P_{Ir}} \end{aligned}$$

18 $$\begin{aligned} & \eta _w =\sum _{i} \eta \ E_{w} \end{aligned}$$

## Performance analysis with inverter current excitations

**Fig. 10:**
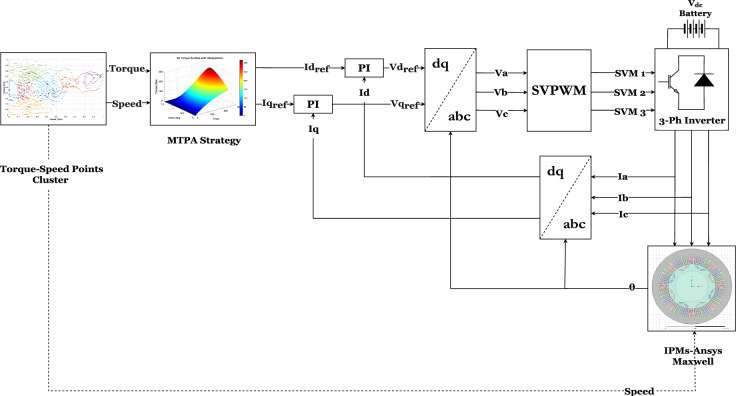
Proposed control schematic for inverter-motor simulation.

This second part presents a comprehensive simulation framework that integrates a closed-loop control system to regulate current excitation in the electric vehicle drivetrain. The system incorporates a three-phase, two-level inverter utilizing Space Vector Pulse-Width Modulation (SVPWM) to generate precise three-phase voltage excitation for the IPMSM, as shown in Fig. 10. The SVPWM technique ensures accurate switching control, improving gate pulse regulation for efficient power delivery. A critical component of this simulation is the current control loop, which ensures precise output current regulation while mitigating the ripple effects induced by the inverter. The d-q axis currents, determined using the MTPA strategy, enable precise current computation, enhancing overall motor performance. Additionally, the inverter introduces transient harmonics into the motor through the output current waveform, further influencing system dynamics. This simulation framework provides an in-depth analysis of motor control behavior, offering valuable insights for improving electric vehicle propulsion systems’ efficiency, stability, and reliability.

This study aims to analyze the impact of inverter harmonics on electric motor performance. A comprehensive dual-model approach is developed in MATLAB, incorporating independent simulations of both IGBT and MOSFET inverters. These inverters utilize a two-level topology operating at a switching frequency of 10 kHz to ensure precise current excitation and reliable motor operation. The command speed in Fig. 10 is derived from the representative operating points obtained via the clustering method applied to the driving cycle data. These points, eight per drive cycle, capture the essential vehicle speed and torque dynamics. For each representative point identified in drive cycles, the simulations are meticulously calibrated to specific values of $i_d$, $i_q$, $L_d$, $L_q$, as detailed in Table 2. This approach ensures an accurate representation of motor behavior across different operating conditions. Each update is carefully implemented to capture the unique characteristics of the machine at every cluster point. Furthermore, the Proportional-Integral (PI) controlller parameters within the control loop, as illustrated in Fig. 10, are dynamically updated in real time. These parameters are determined based on reference^42^, which is based on the motor inductance $L_d$ and resistance $R_s$. This dynamic adaptation ensures that the control system effectively regulates motor performance across varying operational scenarios, enhancing system stability and efficiency.

###  Inverters technical specification

**Fig. 11:**
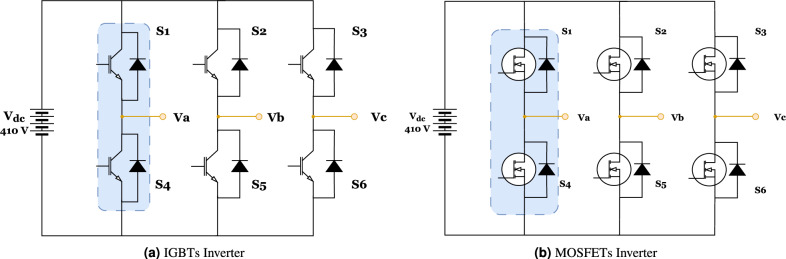
Schematic of the two-level inverter.

Figure 11 illustrates the two types of three-phase voltage source inverters analyzed in this study, highlighting their influence on motor performance throughout the operating cycle. These inverters are essential in electric motor control, converting DC voltage into AC voltage with precise magnitude and frequency to ensure optimal operation under varying conditions. For both inverter models analyzed, a DC input voltage of 410 V was adopted, corresponding to the battery voltage specification of the Bolloré BlueCar electric vehicle.

The first inverter configuration utilizes six IGBTs, recognized for their high power-handling capability and efficiency in medium- to high-power applications. The second type incorporates six MOSFETs, which excel in high-frequency switching due to lower switching losses and faster response times. Both inverter types are meticulously regulated to produce the required AC output, ensuring precise motor control and system stability. By effectively modulating the switching of these semiconductor devices, the inverters generate a well-regulated voltage waveform, minimizing harmonic distortion and enhancing system efficiency. This study investigates how each inverter type impacts key performance parameters, including efficiency, torque ripple, and power losses. The findings provide critical insights into inverter selection for electric vehicle applications, supporting the advancement of power electronics for optimized propulsion systems^43^.

### IGBT and MOSFET inverters model

The SEMiX 603GB12E4SiCp IGBT and SKM 350MB120S-CH17 MOSFET transistors, both manufactured by Semikron, were selected for this study based on their suitability for EV applications, owing to their high efficiency and robust power-handling capabilities. The MOSFET module was particularly chosen for its low switching losses and high-speed switching performance, characteristics that are critical in EV inverter circuits where rapid response and efficient high-frequency operation are required. In this study, the comparison was intentionally conducted under matched conditions to ensure fairness: both inverters use the same rated voltage (1200 V), comparable current ratings (600A and 523A), identical diode energy recovery characteristics ( 0 j)^44^, and an equal switching frequency of 10 kHz, despite the fact that SiC MOSFETs can typically operate at much higher frequencies. This controlled setup allows isolating the influence of device technology on motor–inverter performance without conflating it with differing operating points.

The characteristic data for both the hybrid SiC-based IGBT and full SiC-based MOSFET devices, along with their respective freewheeling diodes, are detailed in Table 5. This information provides key electrical parameters necessary for analyzing inverter performance, particularly with respect to conduction and switching losses. Such analysis is vital for evaluating efficiency, switching behavior, and the overall reliability of the inverter in EV applications. As further highlighted in Table 5 , the SiC-based MOSFET module exhibits markedly lower switching losses compared to the IGBT module, primarily due to its significantly reduced turn-on and turn-off energy dissipation. This underscores the suitability of SiC technology for high-performance, high-frequency applications in EV powertrains.

**Table 5:** IGBT and MOSFET characteristics of the inverter^44,45^.

Parameters	Unit	Modules	
		SEMIX 603GB12E4Sicp	SKM 350MB120SCH17
Inverter type	-	IGBT	MOSFET
Rated voltage ($V_{CES}$)	V	1200	1200
Rated current ($I_C$)	A	600	523
Transistor turn on+off energy dissipation ($E_{on+off}$)	mJ	89	16.6
Transistor collector-emitter threshold voltage ($V_{CE0}$)	V	0.77	0
Transistor on-state slope resistance ($r_{CE}$)	m$\Omega$	2.1	9.5
Diode energy dissipation during reverse recovery ($E_{rr}$)	mJ	0	0
Diode forward threshold voltage ($V_{F0}$)	V	0.8	0.8
Diode on-state slope resistance ($r_{F}$)	m$\Omega$	3.3	4.5

### Inverter losses calculation model

Power semiconductor devices are the primary source of losses in an inverter system. The goal of this test is to find out how much conduction loss there is when the inverter works in both steady-state and continuous modes. We assume that the output currents of all three phases are identical in magnitude and phase for this analysis.

To calculate the losses for a single busbar of the inverter, as depicted in Fig. 11,^13^, it is important to consider the uniformity of the currents. Since the three inverter legs are structurally and functionally identical, the losses calculated for one leg can be extrapolated to the other two. At the same time, as it provides a correct estimate of the overall conduction losses for the whole system, this technique simplifies the computation while reducing its complexity. In general, these losses arise from two mechanisms: conduction losses and switching losses. The conjunction of these two components results in the generation of the total losses seen in power semiconductor devices^46^, which the equation may represent:

19 $$\begin{aligned} P_{\text {inverter}} = 6 \left( P_{\text {con}} + P_{\text {sw}} \right) \end{aligned}$$

This equation symbol $P_{inverter}$ represents the weighted average losses in the power semiconductor device. Furthermore, the notations $P_{con}$ and $P_{SW}$ denote the mean conduction losses and mean switching losses for one semiconductor, respectively. Conduction losses occur when an electric current passes through a semiconductor. Switching losses occur mainly during the transition from the on-state to the off state. During these transitions, the device’s current and voltage display non-instantaneous fluctuations.

####  Conduction losses

To effectively compare conduction losses between IGBT and MOSFET based inverters, it is crucial to accurately evaluate the voltage drops across these components in their respective on-state conditions. This analysis involves assessing the conduction behavior of an IGBT and its reverse diode, as well as that of a MOSFET in a separate circuit. This approach provides a comprehensive yet straightforward representation of the electrical characteristics of both semiconductor devices, facilitating a precise comparison of conduction losses and their impact on overall inverter efficiency.

The total conduction losses in an IGBT inverter model, denoted as $P_{\text {con}}$, comprises two main components: $P_{\text {con,I}}$ and $P_{\text {con,D}}$, as shown in Eq. 20 . These represent the conduction losses associated with the IGBT switch and the freewheeling diode (FWD), respectively. The distribution of these losses is primarily influenced by the direction of current flow during inverter operation. When current flows in the positive direction, forward conduction through the IGBT the resulting conduction losses are attributed to $P_{\text {con,I}}$, which is calculated using Eq. 21. Conversely, when the current reverses and conduction occurs through the FWD, the losses are attributed to $P_{\text {con,D}}$, as defined by Eq. 22,^47–49^ .

20 $$\begin{aligned} & P_{con} = P_{con,igbt\, {\textbf {or}} \, mosfet} + P_{con,D} \end{aligned}$$

21 $$\begin{aligned} & P_{\text {con-igbt}}(t) = \frac{1}{T_{\text {sw}}} \int _{0}^{T_{\text {sw}}} \left( V_{\text {ce0}}(t) .\, i_s(t) + r_c .\, i_s^2(t) \right) .\, dt \end{aligned}$$

22 $$\begin{aligned} & P_{con,D} = \frac{1}{T_{\text {sw}}} \int _{0}^{T_{\text {sw}}} \left( V_{\text {F}}(t) .\, i_s(t) + r_D .\, i_s^2(t) \right) .\, dt \end{aligned}$$

Similarly, in MOSFET-based circuits, conduction losses are primarily determined by the drain-to-source resistance ($R_{on}$) in the on-state. Unlike IGBTs, which exhibit a threshold voltage drop in addition to resistive losses, MOSFET conduction losses are purely resistive and can be expressed as Eq. 23. The magnitude of $R_{on}$ depends on the device characteristics and operating conditions, influencing the overall efficiency of the inverter. By comparing the conduction losses of IGBTs and MOSFETs, it is possible to assess their suitability for different power conversion applications, considering factors such as switching frequency, efficiency, and thermal performance.

23 $$\begin{aligned} & P_{con,mosfet} = \frac{1}{T_{\text {sw}}} \int _{0}^{T_{\text {sw}}} \left( R_{on} .\, i_{s}^2(t) \right) dt \end{aligned}$$

The device datasheet provides detailed information on the key characteristic values required for this analysis^50,51^, in these calculations, $i_s$ represents the stator phase current, while $V_{ce0}$ and $V_F$ correspond to the forward voltage drop across the IGBT and the FWD, respectively, as specified in the device datasheet. The parameter $r_{c}$ denotes the equivalent resistance, which is derived from the voltage-current characteristics of the IGBT and is associated with the collector-emitter voltage ($V_{ce}$).

It is important to note that the conduction losses of the IGBT and the FWD, as expressed in Eqs. 21 and 22, are independent of the switching frequency. This is because conduction losses are primarily determined by the current flowing through the devices and their static electrical properties, such as the forward voltage drop and equivalent resistance, rather than the dynamic behavior associated with switching events.

####  Switching losses

The switching losses in power electronics are mostly determined by two key parameters: the switching energy and the switching frequency of the electronic equipment. The occurrence of these losses during device power state changes may have a substantial influence on the overall efficiency of the system. In the datasheet, the energy required to power the device is generally shown in graphs or tables that illustrate the variations in these energies with various temperatures and currents, as exemplified by the datasheet referenced in this work^50^. During each turn-on and turn-off event, a semiconductor device dissipates energy due to the non-instantaneous nature of the switching transitions. While these transitions are brief in duration, their high repetition rate throughout each second results in cumulative energy losses. As such, total switching losses are directly proportional to the switching frequency^52^.

24 $$\begin{aligned} P_{sw} = P_{{sw,on}\text {(igbt-mosfet)}} + P_{sw,off\text {(igbt-mosfet)}} + P_{sw,off (Diode)} \end{aligned}$$

In the case of IGBT and MOSFET devices, both turn-on $E_{\text {on}}$ and turn-off $E_{\text {off}}$ energy losses must be considered due to their significant contribution to overall switching losses. By contrast, for diodes, only the reverse recovery energy loss $E_{\text {rr}}$ is typically relevant, as turn-on losses are negligible and generally not provided in manufacturer datasheets. In this study, switching loss calculations are performed using interpolation techniques based on empirical data extracted from these datasheets, which typically present energy loss values $E_{\text {on}}$, $E_{\text {off}}$ and $E_{\text {rr}}$ as functions of current at a constant voltage^53^. The inverter’s input is supplied by a DC link voltage $V_{\text {dc}}$, while the reference voltage $V_{\text {ref}}$ plays a critical role in regulating the switching behavior of the inverter. The analytical expressions used to compute switching losses for both IGBT and diode devices are outlined below.

25 $$\begin{aligned} & P_{{sw,on}\text {(igbt-mosfet)}} = \frac{1}{T} \sum _{j=1}^{j=f_\textrm{sw}/f} \left( \frac{I_{out}}{I_{ref}} .\, \frac{V_{dc}}{V_{ref}} .\, E_\textrm{on} \right) \end{aligned}$$

26 $$\begin{aligned} & P_{{sw,off}\text {(igbt-mosfet)}} = \frac{1}{T} \sum _{j=1}^{j=f_\textrm{sw}/f} \left( \frac{I_{out}}{I_{ref}} .\, \frac{V_{dc}}{V_{ref}} .\, E_\textrm{off} \right) \end{aligned}$$

The same method is used to calculate the diode switching loss:

27 $$\begin{aligned} & P_{{sw,off}\text {(D)}} = \frac{1}{T} \sum _{j=1}^{j=f_\textrm{sw}/f} \left( \frac{I_{out}}{I_{ref}} .\, \frac{V_{dc}}{V_{ref}} .\, E_\textrm{err} \right) \end{aligned}$$

28 $$\begin{aligned} & {T = \frac{1}{f}, \qquad \qquad \qquad \qquad f = \frac{P}{2} \cdot \frac{n}{60} } \end{aligned}$$

T: electrical period (seconds), P: number of poles, n: mechanical speed (rpm)

**Fig. 12:**
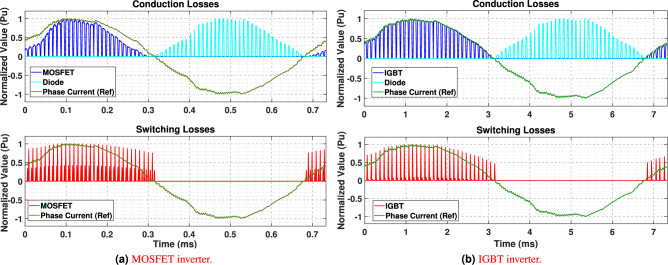
Switching and conduction losses.

The first representative WLTP operating point corresponds to a motor speed of 2048 rpm and a torque demand of 57.3 Nm, for which the MTPA strategy identifies an optimal RMS current of 31.8 A and an electrical phase angle of $\left( \gamma = \text {26.35}^\circ \right)$. Considering the computational complexity of finite element analysis (FEA), the time of simulation is confined to a single electrical period, as illustrated in Eq. 28. Using these excitation parameters, conduction and switching losses were computed for both IGBT- and MOSFET-based inverters. As shown in Fig. 12a and 12b, the phase-A current waveform begins with a non-zero value due to the imposed MTPA phase shift. Switching losses occur only during the turn-on and turn-off transitions of the semiconductor devices, where voltage and current overlap. The diodes do not contribute to switching losses, as their reverse-recovery energy is negligible (Err = 0 j) according to the datasheets. Consequently, the inverter loss evaluation focuses solely on transistor switching losses while comparing the loss distribution of both inverter technologies.

### Proposed methodology

**Fig. 13:**
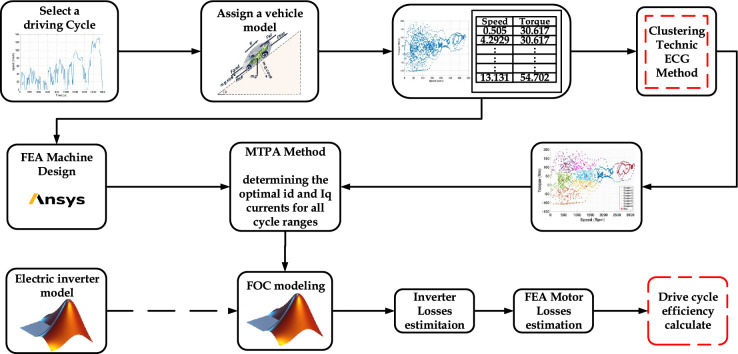
Proposed methodology flowchart.

The overall workflow of the proposed methodology is illustrated in Fig. 13. The process begins with selecting a vehicle model and a target driving cycle, from which the required torque–speed operating profile of the traction electric motor is computed. Because this profile typically contains thousands of points, a clustering procedure is applied to reduce the dataset to a set of representative operating points while preserving the essential dynamics of the original cycle. A suitable IPMSM topology is then defined as shown in Fig. 8, and a flux-map-based modeling approach is used to characterize the machine. This flux map is generated through FEA by scanning the current amplitude and phase (or equivalently $(I_d)$ and $(I_q)$ at a reference speed as shown in Fig. 9). The resulting map enables analytical determination of the optimal current vector for each operating point, using MTPA in the constant-torque region and field-weakening in the constant-power region–while enforcing the inverter’s voltage and current limits. Electromagnetic losses are then evaluated by combining copper losses, which depend on the RMS current magnitude, with speed-dependent iron losses extrapolated from the FEA-derived material loss coefficients. In parallel, a detailed inverter model is used to compute switching and conduction losses for the corresponding MOSFET- or IGBT-based converter. These inverter losses are integrated with the motor losses to obtain the total electro-mechanical losses and to generate combined motor–inverter efficiency maps. This hybrid analytical–FEA procedure enables accurate assessment of motor–inverter performance across the entire driving cycle with substantially reduced computational effort compared to full transient FEA simulations.

## Results

### Evaluate of driving cycles performances without inverter

Based on the analysis in Part Clustering Techniques for Drive-Cycle Data Representation and Performance analysis without inverter the clustering method was used on three standardized driving cycles WLTP, NEDC, and FTP–excluding inverter influences. Table 6 presents a comprehensive examination of motor efficiency, power output, and total power losses at each typical sample point over the whole driving cycle. This method facilitates an efficient and thorough description of driving cycle performances.

A comparison study was performed using the data in Table 7 to confirm the correctness of the clustering algorithm. This assessment used the FEA model of the IPMSM to examine all chosen operating points for each drive cycle, in contrast to the prior technique that depended on a limited number of simulation representative points derived from the clustering method. High-fidelity simulations in Ansys Maxwell accurately represented the motor’s intricate electromagnetic behavior under actual operating circumstances, allowing a precise comparison between the full-cycle analysis and the smaller dataset derived by clustering. The findings validate that the clustering method can effectively depict full-cycle performance while significantly minimizing computing resources.

**Table 6:** Motor efficiency based on different driving cycles clustering.

	Output power (kW)	Total losses (W)	Efficiency	Weighted efficiency
WLTP	12.263	184.54	0.985	0.2563
	-4.207	127.52	0.970	0.0753
	1.117	28.42	0.975	0.0770
	27.581	391.91	0.986	0.2693
	7.074	119.62	0.983	0.1230
	-1.043	32.98	0.969	0.0743
	15.385	374.86	0.976	0.0567
	6.738	320.9	0.954	0.0476
**Drive-cycle motor efficiency: 0.9799**				
NEDC	2.7085	56.5043	0.9794	0.0829
	19.3213	286.1405	0.9853	0.1691
	-4.9887	83.3348	0.9836	0.0223
	-3.3417	114.5887	0.9666	0.0364
	11.0616	176.7496	0.9842	0.2292
	5.3162	243.4869	0.9559	0.0945
	30.2684	448.4754	0.9853	0.1491
	7.3975	143.2164	0.9809	0.1966
**Drive-cycle motor efficiency: 0.9801**				
FTP	8.148	224.54	0.973	0.1139
	5.864	102.69	0.983	0.2347
	7.791	546.18	0.934	0.0880
	0.986	46.98	0.954	0.0429
	-5.353	128.79	0.976	0.0696
	1.956	36.68	0.981	0.2039
	-2.927	213.92	0.931	0.0788
	12.638	188.08	0.985	0.1393
**Drive-cycle motor efficiency: 0.9710**

**Table 7:** Comparison of motor efficiency between All-Points simulation and the clustering method.

Driving Cycle		WLTP	NEDC	FTP
ALL points	Efficiency	0.9661	0.9656	0.9584
	Time	33 h	11 h	61 h
Clustering rps	Efficiency	0.9799	0.9801	0.9771
	Time	10 mn	10 mn	10 mn
	Error	1.38%	1.45 %	1.87 %

As summarized in Table 7, this exhaustive simulation approach reveals important insights into efficiency variation and performance trends, providing a benchmark against which clustering-based evaluations can be assessed. The comparison reveals that while the application of the clustering method introduces a certain level of approximation, the resulting efficiency deviation remains minimal, ranging from 1.38 % in the WLTP cycle to 1.45 % in NEDC and 1.87 % in FTP. Despite this slight loss in accuracy, the clustering method offers a substantial reduction in computational effort, decreasing the full FEA simulation time from 11, 33, and 61 hours for NEDC, WLTP, and FTP cycles, respectively, to approximately 10 minutes per cycle. This demonstrates the method’s effectiveness in significantly accelerating simulations while maintaining acceptable accuracy levels for system evaluation. These results underscore the effectiveness of clustering as a computationally efficient alternative while also highlighting the value of full-cycle simulation for validating reduced-order modeling strategies.

Therefore, this comparison highlights substantial savings in both computational time and resources. Consequently, the remainder of this study adopts a simulation approach based exclusively on RPS collection, eliminating the need to reprocess the complete set of operating points for each analysis.

### Evaluate of driving cycles performances with inverter

#### Influence of inverter excitations on motor performance

**Fig. 14:**
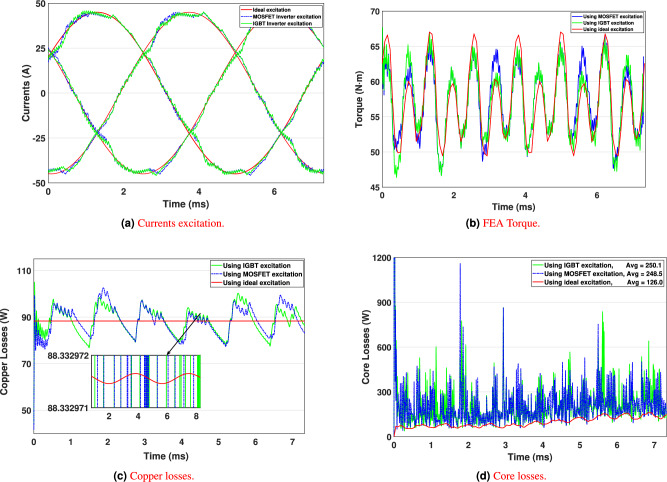
FEA Performance comparison at the first WLTP cluster point of ideal, MOSFET and IGBT simulations.

The simulation results for the output currents $i_a$, $i_b$, and $i_c$ of both the IGBT- and MOSFET-based inverters are illustrated in Fig. 14a, respectively. These figures depict the current waveforms at the first representative load point (RP 1) during the WLTP driving cycle. This specific load point is selected as a reference for further analysis, as the inverter-generated currents are incorporated into the motor model developed in Ansys Maxwell. By integrating these currents, the motor’s performance is evaluated using the FEA under realistic drive cycle conditions.

To validate the accuracy of the motor model, it is essential to achieve the target torque of 57.3 Nm under the given load conditions. Figure 14billustrates the validation of both inverter models, demonstrating their capability to deliver the required excitation for optimal motor torque operation. The results further highlight the effectiveness of the MTPA strategy, which ensures optimal excitation along the d- and q-axes through precise current regulation. This advanced control approach is crucial for maximizing torque generation while minimizing power losses.

The comprehensive analysis of motor response to inverter excitations confirms the accuracy of both the inverter and motor models. Additionally, it underscores the effectiveness of the MTPA strategy in achieving optimal torque performance across various load conditions.

Considering the specified load conditions, the excitation current delivered to the motor inherently contains temporal harmonics introduced by the inverter during operation. These non-ideal current waveforms are imported into ANSYS Maxwell to perform detailed FEA, enabling an in-depth evaluation of how time-domain harmonic content influences overall motor performance.

As depicted in Fig. 14a, the simulation results reveal a significant increase in current ripple for both IGBT- and MOSFET-based excitations relative to ideal sinusoidal waveforms. These findings are consistent with the results shown in Fig. 14b, where the torque production is shown to be highly sensitive to the instantaneous amplitude of current excitation. The result leads to a direct correlation between current ripple, increased torque ripple, and elevated total motor losses, highlighting the critical role of waveform purity in achieving high-performance and energy-efficient electric traction systems.

Figure 14c and 14d present the copper (stranded) and core (iron) losses obtained from the finite element analysis (FEA) simulation for the first load condition. The copper losses remain nearly identical across all load conditions because they primarily depend on the RMS value of the phase current, and the AC copper losses are explicitly included in the FEA computation. In contrast, the ferromagnetic core experiences additional harmonic-dependent losses. As a result, when higher-order harmonics are introduced–such as those generated under MOSFET or IGBT excitation–the core losses increase significantly due to amplified magnetic hysteresis and eddy current effects, explaining the higher energy dissipation observed in these inverter-driven operating conditions.

**Fig. 15:**
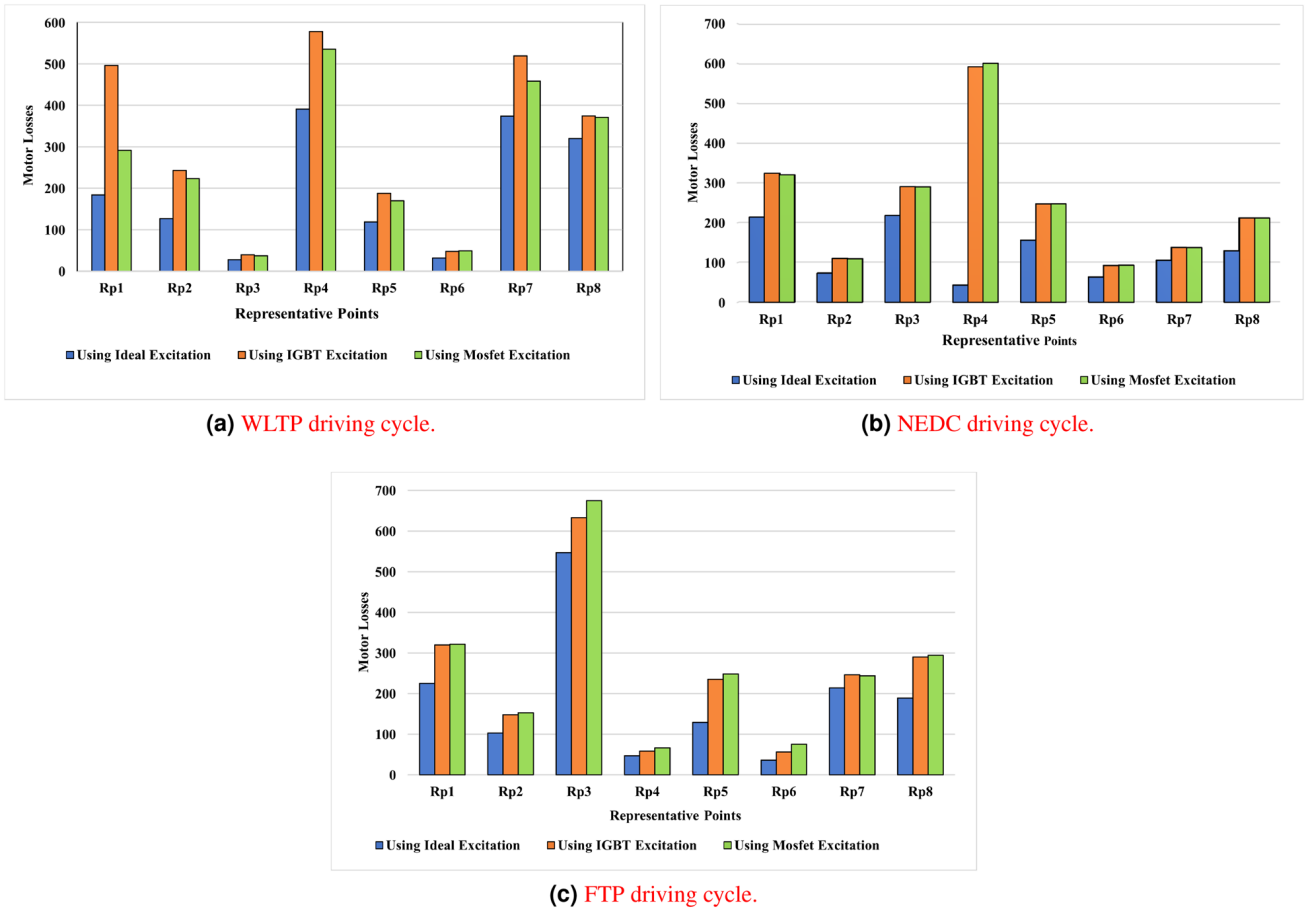
Motor losses over different driving cycles using an ideal excitation source and IGBT and MOSFET inverter excitation devices.

As illustrated in Fig. 15a, 15b, and 15c, the comparative analysis focuses on evaluating motor total losses under three excitation scenarios: ideal sinusoidal excitation, IGBT inverter-derived excitation, and MOSFET inverter-derived excitation. This methodology is consistently applied across representative clustered load points extracted from the WLTP, NEDC, and FTP driving cycles. This approach provides helpful details about the effects of inverter-induced harmonics on motor efficiency, thermal behavior, and overall durability. Furthermore, it emphasizes the influence of inverter topology and current excitation waveform quality in shaping electromagnetic performance, thereby guiding the optimization of electric drive systems.

#### Influence of inverter losses on driving cycle performance

To evaluate the performance of EV in the complete driving cycle, it is essential to account for all categories of power losses associated with both the motor and the inverter. These losses include conduction and switching losses within the inverter, magnetic core losses, and copper losses related to the motor-based inverter excitations, as summarized in Eq. 29. Furthermore, based on the clustering method applied to the driving cycle, the cumulative effect of these losses can be systematically evaluated to quantify efficiency improvements.

29 $$\begin{aligned} \eta = \frac{T_e .\, \omega }{T .\, \omega + P_{cu} + P_{Fe} + P_{inv}} \end{aligned}$$

**Fig. 16:**
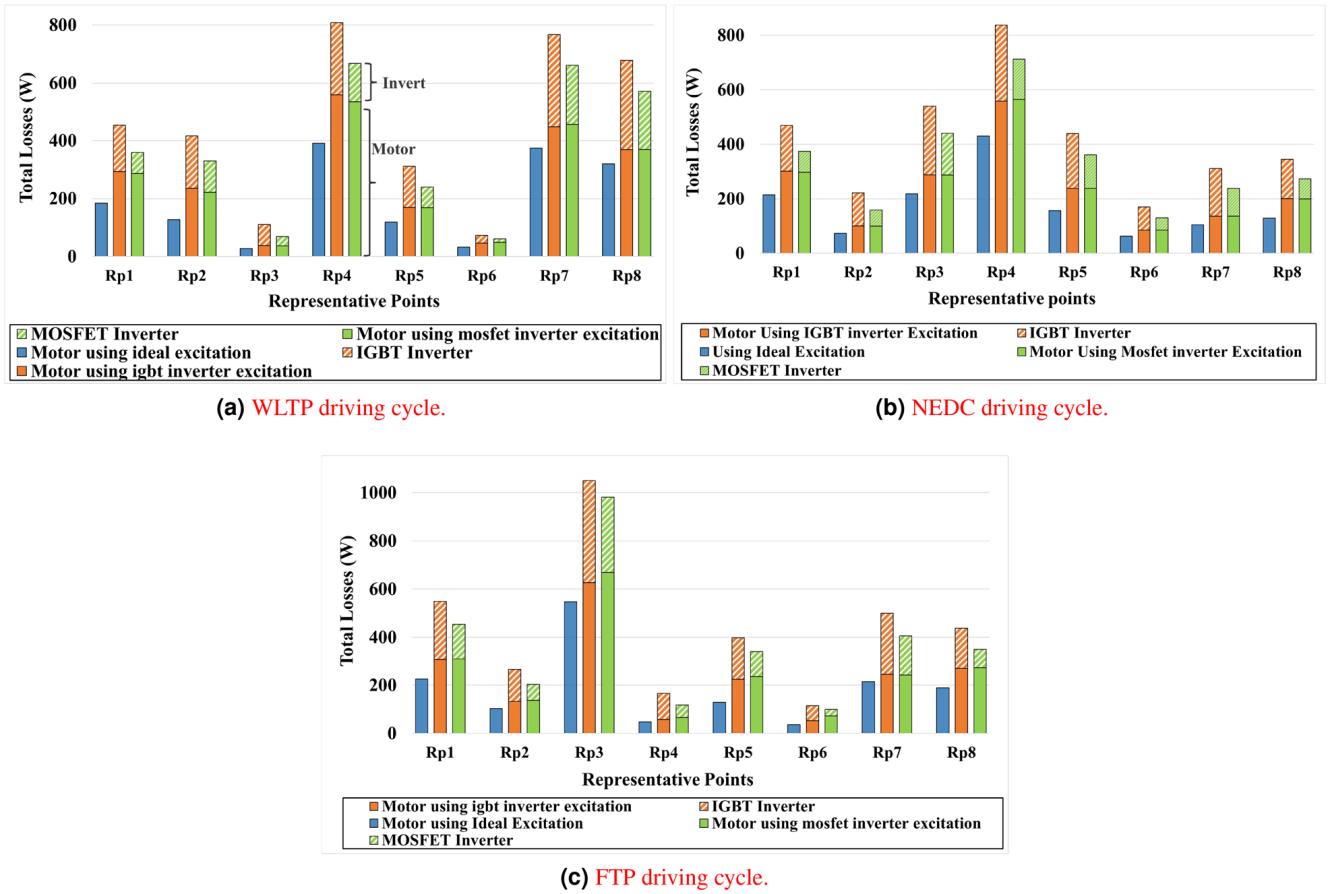
Motor and inverter losses over different driving cycles using an ideal excitation source and IGBT and MOSFET inverter excitation devices.

By integrating all loss components over the representative points of the WLTP, NEDC, and FTP driving cycles, as illustrated in Fig. 16a, 16b, and 16c, this enables an accurate calculation of the overall efficiency of the drivetrain system. This metric is a key indicator for evaluating system performance and energy efficiency. This approach provides a comprehensive representation of total energy consumption, enabling an accurate assessment of the practicality of an electric drivetrain system under realistic operating conditions.

Based on the analysis of the presented Fig. 16a, 16b and 16c it is evident that total loss calculations at any representative point within a driving cycle vary significantly depending on whether the motor operates independently or in conjunction with an inverter, either IGBT or MOSFET-based. For instance, at the first representative point (RP1) of the WLTP driving cycle, the motor-only configuration exhibits losses of approximately 184 W. When the motor is supplied by an IGBT inverter, the motor losses increase to 294 W, and similarly, with a MOSFET inverter, the motor losses are 286.7 W.

However, to accurately determine total power losses in practical scenarios, it is essential to include inverter losses as well. At RP1, the IGBT inverter contributes an additional 159.4 W of losses (including both conduction and switching losses), resulting in a combined total of 453.4 W (294 + 159.4 W). In the case of the MOSFET inverter, the total losses amount to approximately 359.7 W (286.7 + 72.65 W). This comparison clearly demonstrates that, at RP1, the use of an IGBT inverter results in a 246 % increase in total losses relative to the ideal excitation scenario, while the MOSFET inverter yields a 195 % increase. These findings underscore the substantial impact that types of inverter topology have on system-level efficiency and highlight the importance of accounting for inverter-induced losses in electric motor performance evaluations. Based on the demonstrated loss data, Table 8 presents the obtained efficiencies at each representative point across the three driving cycles: WLTP, NEDC, and FTP. These efficiency values reflect the cumulative impact of motor losses and inverter-induced losses, including both conduction and switching components. The table provides a clear comparison between ideal current excitation and inverter-fed excitations (IGBT and MOSFET), highlighting how different inverter technologies influence system performance. This evaluation is essential for understanding how harmonics and switching dynamics affect overall drivetrain efficiency at critical operating conditions representative of real-world driving scenarios.

**Table 8:** Drive cycle efficiency based on different current excitations.

Efficiency	Without Inverter	With MOSFET Inverter	With IGBT Inverter
WLTP	0.9851	0.9714	0.9643
	0.9704	0.927	0.9098
	0.975	0.942	0.9105
	0.9859	0.9763	0.9713
	0.9833	0.9672	0.9577
	0.9689	0.9459	0.9234
	0.9761	0.9588	0.9526
	0.9542	0.9216	0.9085
**Drive cycle** **efficiency**	**0.9799**	**0.9613**	**0.9503**
NEDC	0.9856	0.9751	0.9689
	0.9784	0.9545	0.9377
	0.9453	0.8962	0.8756
	0.9855	0.9764	0.9722
	0.9712	0.9362	0.9234
	0.9834	0.9667	0.9568
	0.9343	0.8635	0.829
	0.9837	0.9664	0.9579
**Drive cycle** **efficiency**	**0.9783**	**0.9572**	**0.9464**
FTP	0.9729	0.9473	0.9369
	0.9826	0.9662	0.9564
	0.934	0.8879	0.8813
	0.9542	0.893	0.8554
	0.9762	0.9401	0.9307
	0.9814	0.9513	0.944
	0.9313	0.8778	0.8538
	0.9851	0.9729	0.9664
**Drive cycle** **efficiency**	**0.9659**	**0.9327**	**0.9214**

**Fig. 17:**
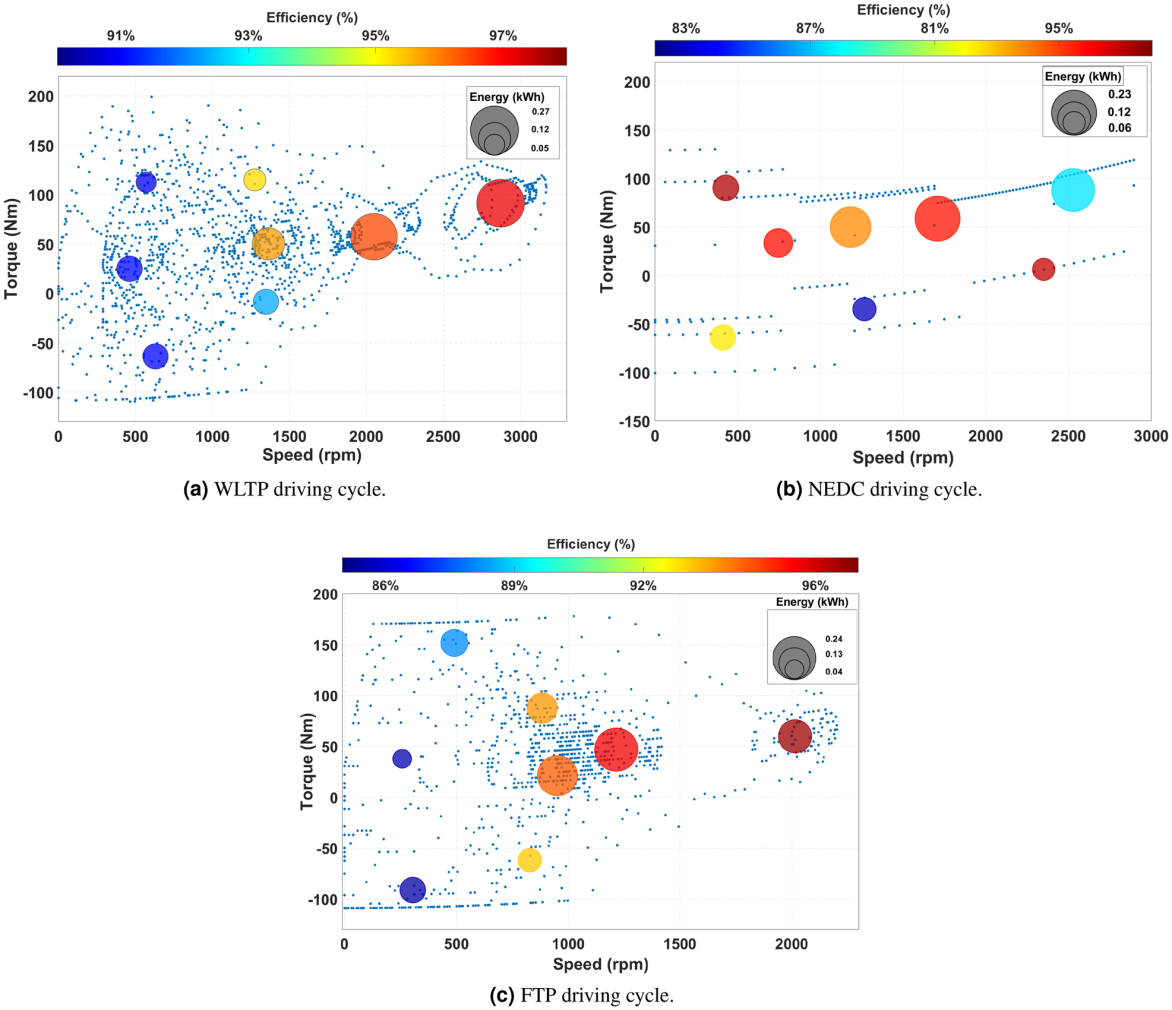
ECG energy distribution and efficiency for different driving cycles with an integrated IGBT inverter.

As illustrated by the data presented in Table 8, the efficiencies at all representative points were evaluated across the WLTP, NEDC, and FTP driving cycles. Under ideal sinusoidal excitation, the average efficiencies were recorded as 0.9799 for WLTP, 0.9783 for NEDC, and 0.9659 for FTP. However, when inverter-induced current excitations were considered, a measurable reduction in efficiency was observed. Specifically, the application of MOSFET inverter excitation resulted in average efficiency reductions of 1.86 %, 2.11 %, and 3.32 % for WLTP, NEDC, and FTP, respectively. Although these reductions indicate the impact of inverter dynamics, the efficiencies under MOSFET excitation remained notably higher than those obtained using IGBT inverter excitation, where the respective reductions reached 2.96 %, 3.19 %, and 4.45 %.

These results, supported by the loss profiles illustrated in the Fig. 16a, 16b and 16c, confirm the substantial influence of inverter-related losses–particularly switching and conduction losses in conjunction with motor losses derived from FEA–on the overall efficiency of the electric drive system. Therefore, the integration of inverter models, including their specific device characteristics, is essential in accurately evaluating electric powertrain performance across real-world driving conditions. This study highlights the necessity of accounting for both the presence and type of inverter when conducting efficiency assessments of electric vehicle systems.

Motor and inverter losses, along with efficiency, are typically illustrated using efficiency maps defined over the torque–speed plane. However, as the present study employs eight representative operating points derived through the ECG clustering method to retain the essential dynamics of the driving cycle while ensuring computational feasibility, constructing a continuous efficiency map is impractical. Instead, Fig. 17a, 17b and 17c present the energy density and efficiency at these representative points, where the motor operates under sinusoidal current excitation generated by the IGBT inverter.

This approach allows inverter losses to be incorporated alongside motor losses in the overall efficiency evaluation, thereby capturing the combined motor–inverter inefficiencies that occur throughout the driving cycle. As a result, the analysis more accurately represents the performance of a realistic electric vehicle powertrain that encompasses the motor, inverter, battery, and gearbox subsystems. Furthermore, by concentrating on these representative points, we can efficiently conduct the optimization process without compromising the fidelity of the full drive-cycle representation. Most of the power distribution is concentrated in the mid- to high-speed range, particularly toward the lower right region of the torque–speed plane.

**Table 9:** Comparison of simulation time.

Driving Cycle	All points Without inverter	Clusterig points		
		Without inverter	With igbt inverter	With mosfet inverter
WLTP	33 h	10 min	62 min	62 min
NEDC	11 h	10 min	37 min	37 min
FTP	61 h	10 min	62 min	62 min

The utilization of inverter models significantly elevates computing demands compared to simulations including only motors, as shown in Table 9. The WLTP cycle required around 10 minutes to use eight typical operating points derived from clustering, without the inverter. The integration of the IGBT or MOSFET inverter models extended the total simulation duration to around 62 minutes in each case. Similar behavior was noted for the NEDC cycle. The simulation duration was increased from 10 minutes (motor only) to 37 minutes depending on each inverter type. During the FTP cycle, calculation time increased from 10 minutes to 62 minutes with the incorporation of inverter dynamics. It is important to acknowledge that these outcomes relate to only eight clustering points.

Simulating the entire driving cycle with all points without an inverter required around 61 hours. The integration of the IGBT or MOSFET inverter models would necessitate a computation two to three orders of magnitude greater, well over 6100 hours, as inverter models require extremely small time steps on the order of $10^{-5}$ s, depending on the switching frequency, rather than the larger steps generally utilized in simulations empty of switching dynamics.

## Conclusion

This paper presents a comprehensive methodology that evaluates the performance of electric vehicles over some standardized driving cycles. The first step consisted of developing a dynamic model of the electric vehicle parameters and then selecting the IPMSM model, which was simulated using finite element analysis in ANSYS Maxwell to precisely model its electromagnetic behavior. Subsequently, three widely recognized driving cycles WLTP, NEDC, and FTP are employed to assess motor efficiency across the full spectrum of real-world operating conditions.

While the full points FEA analysis provides highly accurate results, it requires significant computational time and resources. To address this limitation, the clustering method was applied to identify a reduced set of representative operating points. A comparative analysis between full-cycle simulation and clustering representative points revealed only a small deviation in efficiency results, with a substantial reduction in simulation time.

These findings confirm that the clustering technique offers a practical and efficient alternative for motor performance evaluation. It effectively balances computational cost and accuracy, making it a valuable approach for the design and analysis of EV drivetrains under real-world operating conditions. In addition, the study enhances the accuracy and depth of EV power consumption analysis by incorporating a comprehensive evaluation of losses associated with both the IPMS motor and the inverter converters. The influence of the inverter is particularly significant, as its dynamic behavior directly affects the overall energy efficiency of the EV drivetrain. By accounting for these loss components, the analysis offers a more realistic and precise assessment of the system’s performance under actual operating conditions. Two typical inverters, IGBT and MOSFET, were chosen for this use case, each providing specific electrical attributes that impact system performance. The investigation started with an evaluation of motor losses under ideal excitation conditions, determining a baseline for comparison. While substituting the excitation with actual inverter waveforms, determined by both IGBT and MOSFET switching characteristics, a significant rise in motor losses was observed. This rise is attributed to the presence of high-frequency harmonics and switching ripple in the non-ideal inverter currents, which negatively impact the motor’s electromagnetic performance.

To provide a comprehensive understanding of energy consumption, the inverter switching and conduction losses were computed and included in the motor losses at each typical operating point defined within the WLTP, NEDC, and FTP driving cycles. This incorporation allowed the computation of overall power losses for the whole drivetrain system. After evaluating driving cycle efficiencies, the MOSFET-based inverter consistently reduced power losses more effectively than the IGBT-based configuration. The high-speed switching ability and low switching energy of MOSFETs reduce static losses, making them appropriate for high-efficiency applications with frequent load changes, such as EVs. These results highlight the importance of including combined motor and inverter losses in powertrain methodology and simulation. They emphasize the significance of choosing suitable inverter technology to enhance energy efficiency, thermal performance, and operational lifetime of EV powertrains.

## Data Availability

All data generated or analyzed during this study are fully incorporated within the published article.
